# Downregulation of miR‐33b promotes non‐small cell lung cancer cell growth through reprogramming glucose metabolism miR‐33b regulates non‐small cell lung cancer cell growth

**DOI:** 10.1002/jcb.27961

**Published:** 2018-10-28

**Authors:** Shengping Zhai, Lingyan Zhao, Tiantian Lin, Wei Wang

**Affiliations:** ^1^ Department of Respiratory Yantai Yuhuangding Hospital Affiliated to Qingdao University Yantai Shandong China; ^2^ Department of Thoracic Surgery Yantai Yuhuangding Hospital Affiliated to Qingdao University Yantai Shandong China

**Keywords:** glucose metabolism, lactate dehydrogenase A, non‐small cell lung cancer (NSCLC), microRNAs (miRNAs), miR‐33b

## Abstract

Glucose metabolism is a common target for cancer regulation and microRNAs (miRNAs) are important regulators of this process. Here we aim to investigate a tumor‐suppressing miRNA, miR‐33b, in regulating the glucose metabolism of non‐small cell lung cancer (NSCLC). In our study, quantitative real‐time polymerase chain reaction (qRT‐PCR) showed that miR‐33b was downregulated in NSCLC tissues and cell lines, which was correlated with increased cell proliferation and colony formation. Overexpression of miR‐33b through miR‐33b mimics transfection suppressed NSCLC proliferation, colony formation, and induced cell‐cycle arrest and apoptosis. Meanwhile, miR‐33b overexpression inhibited glucose metabolism in NSCLC cells. Luciferase reporter assay confirmed that miR‐33b directly binds to the 3′‐untranslated region of lactate dehydrogenase A (LDHA). qRT‐PCR and Western blot analysis showed that miR‐33b downregulated the expression of LDHA. Moreover, introducing LDHA mRNA into cells over‐expressing miR‐33b attenuated the inhibitory effect of miR‐33b on the growth and glucose metabolism in NSCLC cells. Taken together, these results confirm that miR‐33b is an anti‐oncogenic miRNA, which inhibits NSCLC cell growth by targeting LDHA through reprogramming glucose metabolism.

## 1 INTRODUCTION

Lung cancer is the first leading cause of cancer‐related deaths in the US. Non‐small lung cancer (NSCLC) constitutes approximately 85% of the cases.^1^, ^2^ Moreover, one‐third of diagnosed patients are suffering from NSCLC of advanced stages (stage III or IV).^3^ NSCLC is the most lethal form of lung cancer and the majority of patients die within first 5 years of diagnosis. The five‐year survival of NSCLC is only 17%.^4^ Recurrence occurs in 30%‐60% of patients with NSCLC.^5^, ^6^ Current treatment options against NSCLC include surgery, adjuvant therapy, chemotherapy, radiotherapy, and immunotherapy. However, effective treatment of NSCLC is still lacking, particularly for advanced stage cancers.^7^ Therefore, it is imperative to develop new therapeutics for this cancer.

MicroRNAs (miRNAs) are small non‐coding RNA of 20 to 24 nucleotides. They bind to the 3′ untranslated region (UTR) of the target gene to induce mRNA degradation.^8^ Altered expression of miRNAs has been observed in different diseases, including cancer.^9^ Recent evidence indicate that miRNAs are important cancer regulatory molecules. One mechanism of this regulation is through the mediation of cancer metabolism. Cancer is known for its unique glucose metabolism to support its rapid growth even under extreme conditions, such as hypoxia. As a result, abnormal glucose metabolism promotes cancer invasiveness and metastasis.^10^, ^11^ It was shown that several miRNAs play a role in glucose metabolism of cancer. For example, miRNA‐195‐5p is known to directly regulate GLUT3 (Glucose Transporter regulator).^12^ miR‐32 have been documented to control the expression of the SLC45A3 protein, which functions as glucose transporter.^13^ miR‐223 is known to upregulate GLUT4 while miR‐133 is known to downregulate its expression.^14^, ^15^ Moreover, miR‐23 indirectly regulates translocation of GLUT4 by regulating SMAD4^16^ and translocation of GLUT4 in adipocytes is regulated by miR‐21.^17^ Several other miRNAs have been reported to regulate the glycolysis. For example, the miR‐143 expression is inversely proportional to the expression of hexokinase 2 (HK2), which mediates aerobic glycolysis.^18^, ^19^ miR‐138 regulates HK1.^18^ One more important mediator of glycolysis in is aldolase A, which was found as a direct target of miR‐122 in liver cells.^20^

Here we study miR‐33b, which was shown as an important tumor suppressor in a variety of cancers, including breast cancer,^21^ osteosarcomas,^22^ colorectal cancer,^23^ and lung adenocarcinoma.^24^ MiR33‐b has been also demonstrated to regulate glucose metabolism.^25^ However, in lung cancer, the role of miR‐33b has not been studied. The aim of our study was to elucidate the role of miR‐33b in NCSLC and study the exact mechanism of this regulation. We found that miR‐33b inhibits the growth of NSCLC through targeting LDHA, thereby reprogramming glucose metabolism. These results could potentiate miR‐33b as a new therapeutic target in NCSLC.

## 2 MATERIALS AND METHODS

### 2.1 Tissue samples and cell lines

Paired non‐small cell lung cancer (NSCLC) and adjacent non‐tumor lung tissues from 22 patients were acquired at Yaitai Yuhuangding Hospital affiliated to Qingdao University. This study was approved by the Ethics Committee of Yaitai Yuhuangding Hospital affiliated to Qingdao University and informed consent was obtained from each patient. Four NSCLC cell lines, A549, SPC‐A1, H1299, and H460, and a normal bronchial epithelial cell line (16HBE) were acquired from the Institute of Biochemistry and Cell Biology of the Chinese Academy of Sciences (Shanghai, China) and cultured in Dulbecco modified Eagle medium (DMEM) supplemented with 10% fetal bovine serum, 100 U/mL penicillin, and 100 mg/mL streptomycin (Invitrogen, Carlsbad, CA) in humidified incubator maintained at 37 ℃ with 5% CO_2_.

### 2.2 Quantitative real‐time polymerase chain reaction

Total RNA was isolated from cells or tissues using the TRIzol reagent (Invitrogen) according to the manufacturer’s protocol. cDNA synthesis and quantitative real‐time polymerase chain reaction (qRT‐PCR) reactions were performed using SYBR Green Assay and the ABI PRISM 7500 Sequence Detection System (ABI). The primers for miR‐33b and lactate dehydrogenase A (LDHA) were 5′‐ATTCTTTCGAACTGTCTTGG‐3′ (miR‐33b, forward), 5′‐TTCACCCTCGGCTGTCCTGACA‐3′ (miR‐33b, reverse) and 5′‐TTGGTCCAGCGTAACGTGAAC‐3′ (LDHA, forward), 5′‐CCAGGATGTGTAGCCTTTGAG‐3′ (LDHA, reverse). U6 snRNA was used as a house‐keeping gene for miR‐33b quantification. qRT‐PCR analyses for LDHA and the normalization control gene GAPDH were performed using SYBR Premix Ex Taq (TaKaRa, Dalian, China). Quantification was performed using the 2^‐△△Ct^ method.

### 2.3 Cell transfection

miR‐33b mimics (5′‐AGGAU CGGUU UGUGCACA‐3′), miR‐33b inhibitor (5′‐AUCGG AUGUG GUGCA CUA‐3′), negative control (NC) (5′‐AUUUGCCAGG UCGGA AUG‐3′) and inhibitor NC (5′‐AGGUC AAGCA GUUCG UUG‐3′) were designed and synthesized by GenePharma (Shanghai, China). Solutions were dissolved in DEPC at a concentration of 20 μM. Approximately 5 to 6 × 10^5^ cells in logarithmic growth phase were seeded in a six‐well plate, followed by adding medium containing serum and double antibody. miR‐33b mimics, NC and serum‐free DMEM were added to cells to a final concentration of 50 nM. miR‐33b inhibitor, inhibitor NC and serum‐free medium were added to cells to a final concentration of 150 nM. For each group, 12 μL of HiPerFect transfection reagent was added to the samples. After incubation at room temperature for 5 to 10 minutes, the cells were added transfection mixture.

### 2.4 Cell proliferation assays

To monitor cell growth, transfected cells were seeded in 24‐well plates (4000 cells per well) in triplicate. At 24, 48, 72, or 96 hours after seeding, the cells were trypsinized and the cell number was counted by a hemocytometer to plot the cell growth curve. In Cell Counting Kit‐8 (CCK‐8, Beyotime) assay, cells were seeded and cultured in 96‐well plates overnight, followed by adding 10 μL of CCK‐8 reagent. After a 2‐hour incubation, the 96‐well plate was placed in a 37°C, 5% CO_2_ incubator, and the absorbance was measured at 450 nm using a microplate reader.

### 2.5 Colony formation assay

Cells were seeded in 12‐well plates at a density of 5900 cells per well. Fresh culture medium added every 3 days. After 7 days, crystal violet staining was performed and the numbers of colonies were counted.

### 2.6 Cell cycle analysis

Cells were collected by trypsinization and washed twice using cold PBS, followed by fixation in 70% ethanol overnight at 4°C. Cells were subsequently incubated with 20 μg/mL propidium iodide (Sigma‐Aldrich) for 20 minutes at room temperature. Cell cycle analysis was performed with FACS flow cytometry (BD Biosciences, Franklin Lakes, NJ).

### 2.7 Detection of lactate production, glucose consumption, and ATP levels

Cells were cultured in DMEM without phenol red for 15 hours. The culture media were then harvested, and the lactate and glucose concentrations were measured using a Lactate Assay kit (BioVisionCA) and glucose assay kit (Sigma‐Aldrich), respectively. ATP levels were quantified using a CellTiter‐Glo^®^ Luminescent Cell Viability Assay (Promega, Madison, WI). Protein concentration, measured using a bicinchoninic acid (BCA) protein assay, were used to normalize all lactate, glucose, and ATP measurements.

### 2.8 Luciferase reporter assay

The 3′‐UTR sequence of wild‐type LDHA and that of a target‐site Mutant (MT) were amplified by PCR, cloned into a dual‐luciferase reporter plasmid (Promega), yielding pGL3‐LDHA–3′‐UTR‐wild‐type (WT) and pGL3‐LDHA‐3′‐UTR‐MT respectively. Cells were inoculated into 96‐well plates at the density of 1.5 × 10^4^ cells per well. Cells were co‐transfected with the WT or MT vector and miR‐33b mimics, NC, miR‐33b inhibitor, or inhibitor NC using the Attractene Transfection Reagent (Qiagen). The ratio of firefly to Renilla luciferase activity was assessed at 48 hours after transfection.

### 2.9 Western blot

Cells lysed in ice‐cold RIPA buffer (Beyotime, China) supplemented with 10 nM PMSF. The protein samples of equal amount were resolved on 10% SDS polyacrylamide gels and transferred to polyvinylidene fluoride membranes at 100 V for 2.5 hours. 5% fat‐free milk in TBST was used to block the membrane, followed by adding primary antibodies (Abcam, Cambridge) (anti‐LDHA, 1: 500) and incubation at 4°C overnight. Secondary antibodies (1:5000) were added and incubated for 2 hours at room temperature. The protein bands were visualized using the chemiluminescence method (Millipore, MA). Image J software (National Institutes of Health, Bethesda) was used to analyze the protein expression levels. GAPDH (1:1000) was used as the control.

### 2.10 Statistical analysis

Statistical analysis was performed using the SPSS 17.0 software. Data were expressed as mean ± SD. The independent‐samples t‐test was used for comparisons between two groups. The one‐way ANOVA test, followed by Bonferroni’s post‐hoc test, was performed to analyze difference among more than two groups. *P* values less than 0.05 were considered significant.

## 3 RESULTS

### 3.1 miR‐33b is significantly downregulated in NSCLC tissues and cell lines

**Figure 1:**
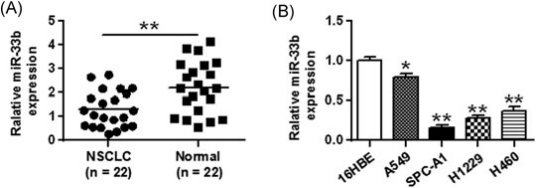
miR‐33b is downregulated in human NSCLC tissues and cell lines. A, The expression levels of miR‐33b were analyzed by qRT‐PCR in 22 pairs of NSCLC tissues and adjacent normal tissues. B, MiR‐33b expression levels in four NSCLC cell lines were measured by qRT‐PCR with snRNA U6 levels as an internal control. *P < 0.05, **P < 0.01. miR, microRNA; NSCLC, non‐small cell lung cancer; qRT‐PCR, quantitative real‐time polymerase chain reaction

The Expression of miR‐33b in 22 NSCLC and adjacent nontumoral normal tissue samples was measured by qRT‐PCR. It was shown that miR‐33b was significantly decreased in NSCLC tissues compared to that in the non‐tumor normal tissues (*P* < 0.01) (Figure 1A). Assessment of miR‐33b expression in four NSCLC cell lines (A549, SPC‐A1, H1299, and H460) and normal human bronchial epithelial cells (16‐HBE) by qRT‐PCR showed that NSCLC cells exhibited significantly lower miR‐33b expression than 16‐HBE cells (*P* < 0.05), with the lowest expression levels detected in SPC‐A1 and H1299 cells (Figure 1B). These two cell lines were therefore used for subsequent functional experiments.

### 3.2 miR‐33b inhibits the growth of NSCLC cells

**Figure 2:**
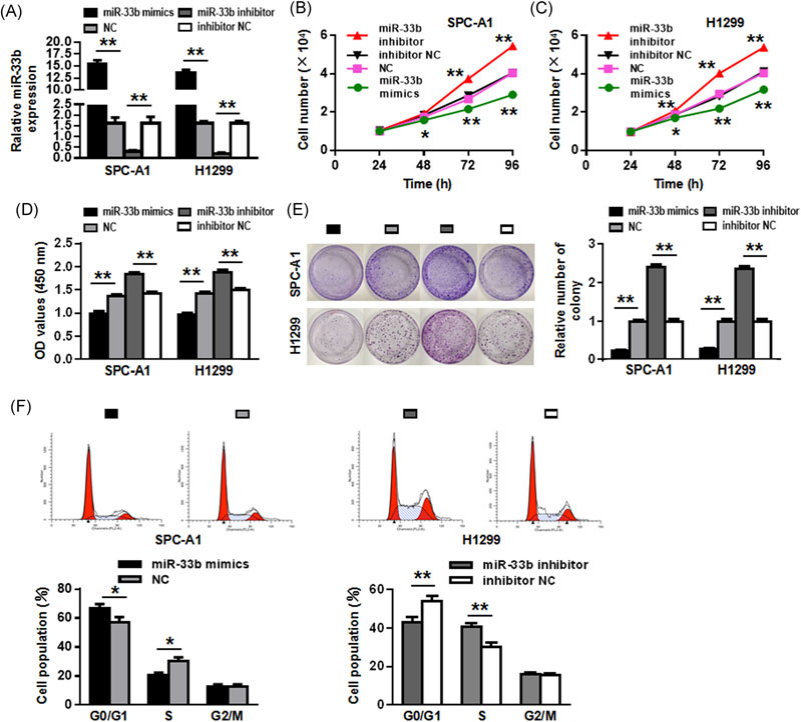
miR‐33b inhibits the growth of NSCLC cells. A, miR‐33b expression levels changed after transfection of SPC‐A1 and H1299 cell lines. B, C, The ectopic expression of miR‐33b significantly suppressed or promoted the cell proliferation of SPC‐A1 and H1299 cells in a time‐dependent manner. D, The results of CCK‐8 assay showed miR‐33b mimics suppressed cell proliferation, and inhibition of miR‐33b leads to enhanced cell proliferation. E, The long‐term proliferative capacity of SPC‐A1 and H1299 cells was detected by colony formation. F, The cell cycle analysis in SPC‐A1 and H1299 cell lines was performed using flow cytometry. *P < 0.05, **P < 0.01. miR, microRNA; NSCLC, non‐small cell lung cancer

We then tested the effect of miR‐33b on SPC‐A1 and H1299 cell growth. Firstly, miR‐33b mimics, non‐coding RNA (NC), miR‐33b inhibitor, and inhibitor NC were transfected into SPC‐A1 and H1299 cells and miR‐33b level were assessed by qRT‐PCR. As shown in Figure 2A, the level of miR‐33b in the miR‐33b mimics group was significantly higher than that in the NC group in both SPC‐A1 and H1299 cells (*P* < 0.01). In addition, miR‐33b expression in the miR‐33b inhibitor group was significantly lower than that in the inhibitor NC group (*P* < 0.01). Moreover, as shown in Figure 2B and 2C, cells transfected with miR‐33b mimics had suppressed cell proliferation, whereas cells transfected with miR‐33b inhibitor showed increased cell proliferation (*P* < 0.05, *P* < 0.01). Consistently, CCK‐8 assay indicated that overexpressing miR‐33b significantly suppressed cell proliferation, and reduction of miR‐33b enhanced that in both SPC‐A1 and H1299 cells (*P* < 0.01) (Figure 2D). We also confirmed the effect of miR‐33b on the long‐term proliferative capacity of SPC‐A1 and H1299 cells, as evidenced in colony formation assay. As shown in Figure 2E, in both NSCLC cell lines, miR‐33b overexpression significantly inhibited colony formation, while reduction of miR‐33b promoted that (*P* < 0.01). Furthermore, flow cytometry was used to analyze the effect of miR‐33b on the cell cycle of NSCLC cell lines. It was shown that upregulation of miR‐33b in SPC‐A1 cells led to a significant increase in the cellular population in G0/G1 phase but a sharp decrease in the S phase, while downregulation of miR‐33b in H1299 cells noticeably induced the opposite effect (*P* < 0.01) (Figure 2E).

### 3.3 miR‐33b regulates glucose metabolism in NSCLC cells

**Figure 3:**
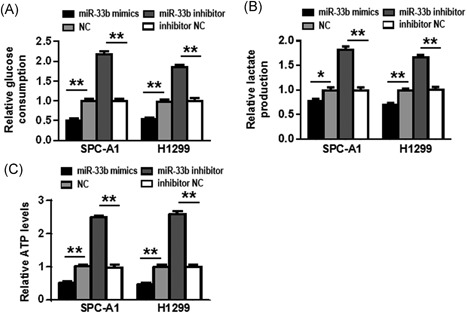
miR‐33b regulates glucose metabolism in NSCLC cells. A‐C, Glucose consumption, lactate production, and ATP levels were detected after SPC‐A1 and H1299 cells were transfected. *P < 0.05, **P < 0.01. miR, microRNA; NSCLC, non‐small cell lung cancer; ATP, adenosine triphosphate

To explore the role of miR‐33b in glycolysis in NSCLC, differences in metabolic parameters were detected in SPC‐A1 and H1299 cells after transfection. We show that upregulation of miR‐33b in SPC‐A1 cells efficiently reduced glucose consumption (Figure 3A), lactate production (Figure 3B) and ATP levels (Figure 3C), while downregulation of miR‐33b increased these metabolic parameters (*P* < 0.01). The similar results were also observed in another NSCLC cell line, H1299 cells (*P* < 0.01) (Figure 3A‐C).

### 3.4 LDHA is a direct target of miR‐33b

**Figure 4:**
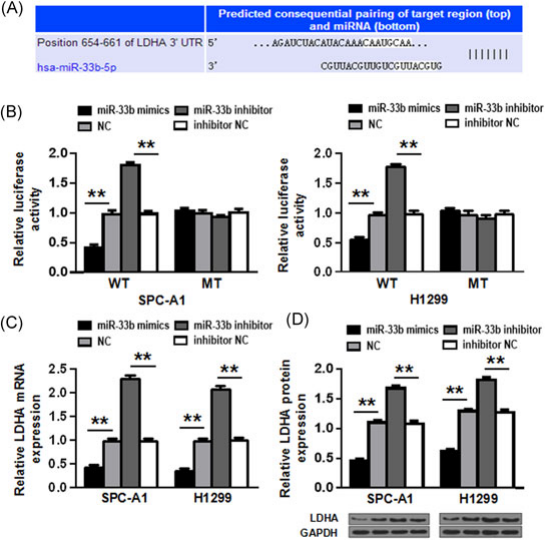
LDHA is a direct target of miR‐33b. A, Predicted binding between miR‐33b and the seeds matched in the 3′‐UTRs of LDHA. B, Luciferase assay in SPC‐A1 and H1299 NSCLC cells. C, LDHA mRNA levels were analyzed after miR‐33b transfection by qRT‐PCR. D, miR‐33b transfection affects LDHA protein levels. **P < 0.01. miR, microRNA; NSCLC, non‐small cell lung cancer; LDHA, lactate dehydrogenase A; qRT‐PCR, quantitative real‐time polymerase chain reaction

To elucidate the mechanism of miR‐33b regulation in NSCLC, we utilized bioinformatics analysis and identified that LDHA might be a putative target gene of miR‐33b (Figure 4A). To corroborate this, we explored whether miR‐33b had a functional role in regulating LDHA expression. LDHA WT or MT 3′‐UTR was subcloned into a luciferase reporter vector, followed by co‐transfection with miR‐33b mimic or miR‐33b inhibitor into SPC‐A1 and H1299 cells. It was shown that luciferase activity in miR‐33b mimic was significantly lower than that of cells transfected NC in the LDHA‐3′UTR‐WT group (*P* < 0.01), but that was no significant difference in the LDHA‐3′UTR‐MT group (*P* > 0.05), while luciferase activity was significantly higher in miR‐33b inhibitor than that for cells transfected with inhibitor NC in the LDHA‐3′UTR‐WT group (*P* < 0.01), and luciferase activity showed no significant difference in the LDHA‐3′UTR‐ MT group (*P* > 0.05) (Figure 4B). Furthermore, overexpression of miR‐33b significantly downregulated LDHA mRNA and protein levels, while reduction of miR‐33b significantly upregulated that in both NSCLC cell lines (*P* < 0.01) (Figure 4C**, D**).

### 3.5 LDHA expression attenuates the growth inhibitory effect of miR‐33b on NSCLC cells

**Figure 5:**
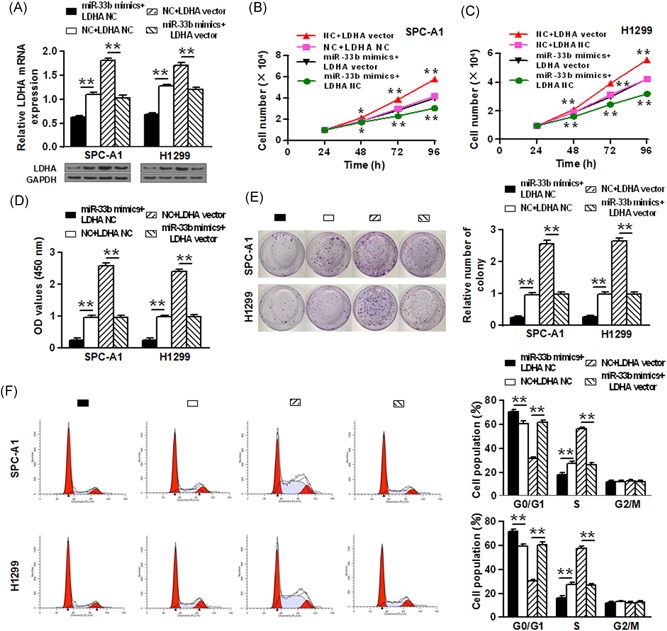
LDHA expression attenuates the growth inhibitory effect of miR‐33b on NSCLC cells. A, LDHA vector attenuated the inhibitory effect of miR‐33b mimics on LDHA protein. B‐D, The results the of the growth curve and CCK‐8 assays showed LDHA vector attenuated the inhibitory effects of miR‐33b mimics on cell proliferation of SPC‐A1 and H1299 cells. E, The long‐term proliferative capacity of SPC‐A1 and H1299 cells was detected by colony formation. F, The cell cycle analysis in SPC‐A1 and H1299 cell lines was performed using flow cytometry. *P < 0.05, **P < 0.01. miR, microRNA; NSCLC, non‐small cell lung cancer; LDHA, lactate dehydrogenase A

To further determine the role of LDHA in miR‐33b‐regulated NSCLC cells, we designed an LDHA vector and co‐transfected SPC‐A1 and H1299 cells with this vector and miR‐33b mimics. It was found that the LDHA vector attenuated the inhibitory effect of miR‐33b mimics on LDHA protein in NSCLC cells (*P* < 0.01) (Figure 5A). The LDHA vector also attenuated the growth inhibitory effect of miR‐33b on NSCLC cells (*P* < 0.05, *P* < 0.01) (Figure 5B‐F).

### 3.6 LDHA expression attenuates the inhibitory effect of miR‐33b on glucose metabolism in NSCLC cells

**Figure 6:**
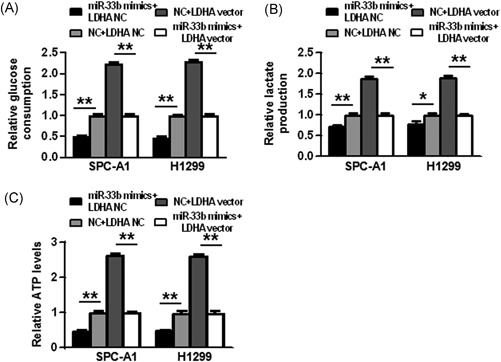
LDHA expression attenuates the inhibitory effect of miR‐33b on glucose metabolism in NSCLC cells. A‐C, Glucose consumption, lactate production, and ATP levels were detected after SPC‐A1 cells were transfected. D‐F, Glucose consumption, lactate production, and ATP levels were detected after H1299 cells were transfected. *P < 0.05, **P < 0.01. miR, microRNA; NSCLC, non‐small cell lung cancer; ATP, adenosine triphosphate

Finally, we explored whether miR‐33b could inhibit the glycolysis of NSCLC by targeting LDHA. We show that glycolysis‐suppressing effects induced by miR‐33b could be reversed by LDHA overexpression, as evidenced by increased glucose consumption, lactate production and ATP levels after LDHA vector transfection (*P* < 0.05, *P* < 0.01) (Figure 6).

## 4 DISCUSSIONS

Treatment of NSCLC still remains a challenge. Currently, surgery is the primary treatment modality for NSCLC patients in stage I, II &III. Chemotherapy is normally given to the patients suffering from stage IV cancer. However, the therapeutic outcome of these treatments is still suboptimal. The use of miRNAs as regulatory molecules of lung cancer is emerging. Here we show that miR‐33b is downregulated in NSCLC tissue and cells, which is in line with previous reports that miR‐33b serves as a tumor suppressor.^26^, ^27^ Further, we showed that miR‐33b overexpression attenuated the proliferation, colony formation of NSCLC cells and promoted cell cycle arrest and apoptosis (Figure 2). These data implicated the use of miR‐33b for NSCLC therapy. Indeed, recent evidence identified a number of miRNAs as novel NSCLC suppressors, e.g miR‐513a‐3p, miR‐200b, miR‐100, let‐7c, miR‐101, miR‐186, miR‐34ac, miR‐24, and miR‐148a, or promoters, e.g miR‐21, miR‐135a, miR‐30c, and miR‐100.^28^ Overexpression or suppression of these molecules was shown to effectively attenuate NSCLC progression. For example, Zhang et al showed that miR‐143 targets epidermal growth factor receptor (EGFR) and suppresses the cell proliferation and invasion of NSCLC in vitro, suggesting miR‐143 as potential therapeutic target against NSCLC.^29^ Therefore, our study underscored the critical role of miRNAs in cancer and miRNAs other than miR‐33b may also be important regulators of NSCLC. It is also worth noting that our study is limited to in vitro analysis of the cell physiologies affected by miR‐33b, and it is imperative to validate the therapeutic potential of miR‐33b in vivo.

Despite recent advances in the understanding of miRNA regulation in cancer, investigation of the role of miRNAs in cancer metabolism, whereas, is rarely studied. Our study is preceded by evidence showing that miRNAs participate in the regulation of glucose and fatty acid metabolism in other diseases.^30^ Cancer are characteristic of elevated glucose metabolism, a mechanism that has been utilized in imaging detection of cancer, such as the 18F‐FDG PET.^31^ Here we for the first time showed that miR‐33b attenuates glucose metabolism in NSCLC. When the SPC‐A1 and H1299 NSCLC cells were transfected with miR‐33b, significantly reducing of the metabolic parameters, including consumption of glucose, ATP and lactate production was seen. In line with this, downregulation of miR‐33b by using its inhibitor results in increased glucose consumption, ATP levels and lactate production within cells.

Further, as a mechanistic study, we identified LDHA as a target of NSCLC. LDHA is an enzyme of the glycolytic pathway, which plays a role in the regulation of glycolysis in anaerobic conditions. LDHA has been used as a biomarker in various cancers.^32^ In our study, the interaction between LDHA and miR‐33b was verified by luciferase reporter assay. Moreover, overexpression of LDHA was shown to antagonize the tumor‐suppressing effects of miR‐33b. Several signaling pathways have been previously identified to regulate LDHA, including JMJD2A‐LDHA (Jumonji C domain 2A‐Lactate dehydrogenase A) and KLF4/LDHA (Krüppel‐like factor 4‐ Lactate dehydrogenase A) pathways, which are crucial to glycolysis regulation in cancer.^33^, ^34^ Zhao et al demonstrated the inhibitory role of miR‐33b in malignant melanoma by regulating LDHA.^26^ Notably, miR‐33b also regulates the metabolism of fatty acids,^35^ and other cancer‐related processes, such as angiogenesis, hypoxia, etc., which presumably all contribute to the tumor‐attenuation effects of miR‐33b.^26^, ^27^ Besides, as a broad‐spectrum regulatory molecules, miR‐33b has also been shown to regulate genes, such as NPC1 (Niemann Pick C), ABCA1 (ATP‐Binding Casette A1), ABCG1 (ATP‐Binding Casette G1), CPT1A (carnitine palmitoyltransferase 1A), CROT (carnitine O‐octanoyltransferase), and HADHB (hydroxyacyl‐CoA dehydrogenase/3‐ketoacy‐CoA thiolase/enoyl‐CoA hydratase *β* subunit).^36^ These factors should also be considered to maximize the therapeutic utility of miR‐33b.

## 5 CONCLUSIONS

In this study, it has been found that in NSCLC cells/tissue the miR‐33b is significantly downregulated. Moreover, miR‐33b overexpression not only inhibits the growth of NSCLC cells but also attenuates the glucose metabolism. The rate of glucose metabolism is inversely proportional to the level of miR‐33b. More miR‐33b expression results in a decreased rate of glucose metabolism. The study also found that inhibition of NSCLC cells growth by miR‐33b may be regulated through targeting LDHA. It has also been found that miR‐33b inhibits the glucose metabolism in NSCLC cells by targeting LDHA. In short, miR‐33b acts as an anti‐NSCLC molecule by reprogramming glucose metabolism through targeting LDHA.

## CONFLICTS OF INTEREST

The authors declare that they have no conflicts of interests.

## AVAILABILITY OF DATA AND MATERIALS

The analyzed data sets generated during the study are available from the corresponding author on reasonable request.

## ETHICS APPROVAL AND CONSENT TO PARTICIPATE

The current study was approved by the Ethics Committee of the Yaitai Yuhuangding Hospital affiliated to Qingdao University. All patients and healthy volunteers provided written informed consent before their inclusion within the study.

## CONSENT FOR PUBLICATION

All authors have read and approved the final manuscript.
